# Use of ginger extract and bacterial inoculants for the suppression of *Alternaria solani* causing early blight disease in Tomato

**DOI:** 10.1186/s12870-024-04789-z

**Published:** 2024-02-21

**Authors:** Sajjad Hyder, Amjad Shahzad Gondal, Anam Sehar, Aimen Razzaq Khan, Nadia Riaz, Zarrin Fatima Rizvi, Rashid Iqbal, Mohamed S. Elshikh, Khaloud M. Alarjani, Muhammed Habib ur Rahman, Muhammad Rizwan

**Affiliations:** 1 Department of Botany, Government College Women University Sialkot, Sialkot, 51310 Pakistan; 2 Department of Plant Pathology, Bahauddin Zakariya University, Multan, 60000 Pakistan; 3 Directorate of Student Affairs and Student Counselling Service – SA&C, Lahore Garrison University Lahore, Lahore, 54000 Pakistan; 4 Department of Botany, Lahore College for Women University, Lahore, 54000 Pakistan; 5 Department of Agronomy, Faculty of Agriculture and Environment, The Islamia University of Bahawalpur, Bahawalpur, 63100 Pakistan; 6 Department of Botany and Microbiology, College of Science, King Saud University, P.O. 2455, 11451 Riyadh, Saudi Arabia; 7 Institute of Crop Science and Resource Conservation (INRES), University of Bonn, Bonn-53115, Germany; 8 Present Address: Department of Seed Science and Technology, Institute of Plant Breeding and Biotechnology (IPBB), MNS-University of Agriculture, Multan-66000, Pakistan

**Keywords:** *Alternaria solani*, Ginger extract, Bacterial inoculants, Early blight disease, Plant growth promotion, Tomato crop

## Abstract

Early blight (*EB)*, caused by *Alternaria solani*, is a serious problem in tomato production. Plant growth-promoting rhizobacteria promote plant growth and inhibit plant disease. The present study explored the bio-efficacy of synergistic effect of rhizobacterial isolates and ginger powder extract (GPE) against tomato *EB* disease, singly and in combination. Six fungal isolates from symptomatic tomato plants were identified as *A. solani* on the basis of morphological features i.e., horizontal septation (6.96 to 7.93 µm), vertical septation (1.50 to 2.22 µm), conidia length (174.2 to 187.6 µm), conidial width (14.09 to 16.52 µm), beak length (93.06 to 102.26 µm), and sporulation. Five of the twenty-three bacterial isolates recovered from tomato rhizosphere soil were nonpathogenic to tomato seedlings and were compatible with each other and with GPE. Out of five isolates tested individually, three isolates (St-149D, Hyd-13Z, and Gb-T23) showed maximum inhibition (56.3%, 48.3%, and 42.0% respectively) against mycelial growth of *A. solani*. Among combinations, St-149D + GPE had the highest mycelial growth inhibition (76.9%) over the untreated control. Bacterial strains molecularly characterized as *Pseudomonas putida*, *Bacillus subtilis*, and *Bacillus cereus* and were further tested in pot trials through seed bacterization for disease control. Seeds treated with bacterial consortia + GPE had the highest disease suppression percentage (78.1%), followed by St-149D + GPE (72.2%) and Hyd-13Z + GPE (67.5%). Maximum seed germination was obtained in the bacterial consortia + GPE (95.0 ± 2.04) followed by St-149D + GPE (92.5 ± 1.44) and Hyd-13Z + GPE (90.0 ± 2.04) over control (73.8 ± 2.39) and chemical control as standard treatment (90.0 ± 2). Ginger powder extracts also induce the activation of defence-related enzymes (TPC, PO, PPO, PAL, and CAT) activity in tomato plants. These were highly significant in the testing bacterial inoculants against *A. solani* infection in tomato crops.

## Introduction

Tomato (*Lycopersicon esculentum* Mill.) is an economically significant crop sown worldwide. After potatoes, it is known to be the second most consumable crop [1]. In the year 2018, around 182 MT of tomatoes were grown in an area of 4.76 MH in more than 150 countries [2]. According to the Government of Punjab, In 2017–18, a total of 414,645 tonnes of tomatoes were produced, covering an area of 41,731 hectares. In 2017–18, it was grown on 8274, 24,968, 5354, and 3135 hectares in Punjab, Sindh, Baluchistan, and Khyber Pakhtunkhwa (KP), with respective production totaling 109,445, 182,198, 37,556, and 85,446 tonnes [3]. Tomato is a very nutritious crop and is considered an important constituent of a balanced diet due to the existence of the high amount of different vitamins like Vitamin A, B & C and some minerals[2, 4]. Tomato exhibits antimicrobial and radical scavenging activity that may help to fight against carcinogenic compounds [5].

One of the most significant constraints in tomato production is diseases. It lowers the product's quantity along with its commercial worth. Tomato plants are threatened by a variety of devastating diseases due to viruses, nematodes, bacteria, and fungi [6]*.* Fungal infections are far more prone to cause significant harm [7]. Solanaceae family members, including tomato, eggplant, pepper, and potato are susceptible to many phytopathogenic fungal strains[8]. *Alternaria solani*, a phytopathogenic fungus, causes *EB* disease in tomato plants, damaging tomato crops by reducing crop yield by about 50% worldwide [9].

The quality and quantity of tomato production have been declined by means of several pests and diseases, respectively [10]. Furthermore, the *Alternaria* fungus could lead to disease in all plant parts (stem collar rotting, leaf blight, and lesions in fruits), causing serious harm at any phase of growth [11]. *Alternaria solani* is an air-born soil hindering fungus that mainly causes destructive yield loss in crops, approximately 80% yield loss has been accounted in tomato crops [4]. The most common devastating *EB* tomato disease (*A. solani*), mainly showed utmost symptoms on the stem, foliage, and fruits, leading to the severity of defoliation, affecting the photosynthetic rate, stunted growth, and loss of yield, respectively [12, 13]. In order to, overcome this problem chemical methods have been used, mainly expensive fungicides, not considered a long-lasting solution, and the most important concern is not appropriate for environmental and public health as well as responsible for fungicide resistance development in *A. solani* [4, 14, 15]. In contrast, researchers found alternative modified modern biocontrol methods to suppress the activity of pathogens. Many studies have revealed that biocontrol technologies are not only environmentally friendly, long-term useful, effective against diseases, and healthy, but they also considerably improve the quality and quantity of tomato crop yield output [14].

Plants act as major natural elicitors against pathogens and rhizospheric zone associated with the variety of microbiomes, which can help in plant growth, stress tolerance, and control of phytopathogens. Plant products and biocontrol agents are ecofriendly and have potential against a wide range of plant infections. Several plant species explored for natural compounds that are effective against phytopathogenic fungi [16]. Recently, many studies with some modifications revealed that plant extracts along with PGPR have been considered the utmost strategy for the management and control of diseases [17, 18]. Ginger (*Zingiber officinale*) show antifungal activity against various microbes due to the presence of monoterpenoids, sesquiterpenoids, phenolic compounds, and its derivatives, aldehydes, ketones, alcohols, esters, which make it an interesting alternative to synthetic antimicrobials [19, 20]. Recent studies have reported the antifungal activities of ginger extract and ginger essential oil against *Fusarium oxysporum* and *Colletotrichum falcatum* respectively [21, 22].

Similarly, investigation and evaluation of beneficial PGPR strains along with some plant extracts, composting, and biochar amendments are used to enhance soil fertility and suppressed the pathogenic mechanism [2], despite all PGPR being useful for the enhancement of crop yield and improvement both qualitatively and quantitatively [4, 23]. Amongst the novel and innovative organic charcoal-like products biochar obtained from a raw organic source (such as green waste, wood chips, poultry manure, etc.) has revealed significant and promising effects against many phytopathogen [24]. Moreover, mycorrhizal fungi are also extensively used to suppress the disease mechanism of *A.solani* and provide protection from the destructive loss of tomato crops [25].

PGPR not only work against infections but also stimulates growth regulators, boosting the quality of crops. They support plant growth and reduce *EB* disease incidence in tomato crops against *A. solani* [2]. PGPR produces HCN, siderophores, and P-solubilizing enzymes that stimulate plant development [26, 27]. *Bacillus* and *Pseudomonas* spp. improves disease resistance and plants. The use of bacterial inoculants is a useful tool for managing plant dieases [28]. PGPR are widely present in agricultural soil and display important properties which make them efficient but their antagonistic potential against the *EB* of tomatoes particularly from Pakistan has not been studied extensively. The current study was designed to evaluate the antimicrobial activity of PGPR alone and combined with GPE against *A. solani* under in vitro conditions and pot trials.

## Materials and methods

### Collection of diseased plants and pathogen isolation

During a field survey in December 2020, infected leaves showing early blight symtoms were seen and purchased from a local market there in zipped bags from J.K Forms and markets, Faisalabad, Pakistan. All the collected samples were transferred to the Department of Botany, GC Women University Sialkot, Pakistan, and were stored at 4 °C until further use. The collected samples were processed for the isolation of *Alternaria* sp. by following the method reported by Babu et al. [4]. Leaf samples were cleaned under running tap water to remove all the soil particles. The infected leaves were chopped down into small segments, surface sterilized with a 0.5% NaOCl olution for 2–3 min, washed thrice with sterile distilled water, dried on 3-layered blotter paper, and plated on PDA containing Petri dishes under aseptic conditions. Petri dishes were incubated at 26 °C for 5–7 days. The growing fungus was further purified on PDA media and stored at 4 °C until further use in experiments.

### Identification and morphological characterization of *A. solani*

*A. solani* was identified microscopically by comparing it with the morphological features already reported [29, 30]. The morphological features including colony color, colony margins, mycelial growth, horizontal and vertical septation, sporulation, length and width of conidia, and beak length of three type fungal isolates representing different locations (*AS-1, AS-3*, *and AS-5*) were observed under a light microscope (40X power lens).

### Pathogenicity assay

The pathogenicity assay of six *A. solani* isolates was performed on a susceptible tomato (variety; Rio Grande) by performing detached leaf assay previously reported by Babu et al. [4]. In brief, 7 days old culture of *A. solani* grown on potato dextrose agar (PDA) media was flooded with sterile distilled water (SDW) to prepare a conidial suspension. The conidial load in the suspension was maintained at 5 × 10^4^ conidia ml^−1^ by using haemocytometer. Randomly, 10 healthy leaves from 4-week old tomato seedlings were collected, washed with tap water, followed by rinsing with SDW, blot dried, and placed on the wet blotter paper on the Petri plates in three replicates. All the collected leaves were covered with wet blotter on the upper lids of the Petri plates. The leaves were injected with 50 µL conidial suspension of *A. solani* at the center while the leaves treated with 50 µL SDW served as control treatments. All the treatments were kept at 25 ± 2 °C, monitored for three weeks for disease symptoms development. Observations on disease severity were taken by following disease rating scale; 0 = no lesions on leaflets, 1 = 1–10% leaf area damaged, 2 = 11–25% leaf area damaged, 3 = 26–50% leaf area damaged, 4 = 51–75% leaf area damaged, and 10 = 100% symptoms on tomato leaves. *A. solani* isolate depicting the highest disease severity was selected to use in further experiments.

### Isolation of rhizobacteria

For the isolation of bacterial strains, rhizospheric soil was sampled from the healthy tomato fields located at JK Agriculture Farm Faisalabad, Pakistan. Rhizobacteria were isolated by following the procedure of Hibar et al. [31]. In brief, 1 g rhizospheric soil was serially diluted in distilled water and dilutions from 10^–2^ to 10^–7^ were prepared. Afterward, 0.1 ml aliquot was spread on solidified NA media (give full form of media) and placed at 26 ± 2 °C for 48 h.

### Compatibility among rhizobacterial strains

The methodology of Fukui et al. [32] was followed to study the compatibility among the rhizobacterial strains. In repeated experiments, bacterial strains were cross streaked on the same NA containing Petri plates in triplicate followed by incubation at 26 ± 2 °C for 72 h. Bacterial growth inhibition data was taken from each treatment for 48 and 72 h of incubation.

### Preparation of GPE

Fresh, plump ginger roots with smooth skin and few creases were washed and grated followed by sun drying. Spice grinder was used to pulverize the dried ginger and powder was kept cold in dark. The extract was made by mixing a small amount of ginger powder with a ratio of 1 part ginger powder to 9 parts water.

### Compatibility of GPE with rhizobacterial isolates

The compatibility of GPE with bacterial strains viz, Hyd-01F, Hyd-13Z, Gb-T23, St-149D, and Ft-G43 was tested on NA medium in a repeated experiment. In this assay, 48 h-old bacterial cultures were spread on solidified NA plates. Three small paper discs (5 mm) were placed at equidistance to each other on each petri plate. After this, 10 µL of the 10%, 15%, and 20% GPE were loaded to sterile filter paper discs. Similarly, discs treated with 10 µL of 100 ppm streptomycin sulfate and dH_2_O served as control treatments. All the plates were incubated at 26 ± 2 °C for 48 h and development of inhibition zones around the discs were observed. Halo zones formation indicated the incompatibility between the GPE and bacterial strains while the absence of inhibition zones confirmed the compatibility [32].

### *In-vitro* anti-mycotic efficacy of individual and combined application of rhizobacteria and GPE

The dual culture methodology [33] was followed to test the efficacy of individual and combined application of bacterial strains (Hyd-01F, Hyd-13Z, Gb-T23, St-149D, and Ft-G43) and GPE on mycelial growth of *A. solani in-vitro*. For this, sterilized filter paper discs (5 mm) were placed on one side of the PDA containing Petri plates. Discs were treated with 6 µL of 48 h old bacterial strains and GPE individually. For combined applications, 3 µL of individual bacterial strain and GPE was placed on the sterilized filter paper discs aseptically. Actively growing mycelial plugs (9 mm) from 7 day old *A. solani* culture were placed on the other side of the plate. The control treatments contained filter paper discs spotted with distilled water only. Plates were kept at 26 ± 2 °C for 7 days. The repeated experiments were performed with three repeates for each treatment. Data on fungal mycelial growth inhibition % was taken from each treatment by given formula:

$$\mathrm M\mathrm y\mathrm c\mathrm e\mathrm l\mathrm i\mathrm a\mathrm l\;\mathrm G\mathrm r\mathrm o\mathrm w\mathrm t\mathrm h\;\mathrm I\mathrm n\mathrm h\mathrm i\mathrm b\mathrm i\mathrm t\mathrm i\mathrm o\mathrm n\;\%=\frac{\text{C}-\mathrm T}{\text{C}}\mathrm x100$$

Where C = Mycelial growth in Control (*cm*); T = Mycelial growth in Treatment (*cm*).

### Biochemical assay

Various biochemical assays were performed for the identification of PGPR. The Gram staining assay was performed by following the procedure of Vincent and Humphrey [34] while the KOH solubility assay was carried out following the methodology as described by Kirsop and Doyle [35]. Gram staining was performed by employing crystal violet, iodine, ethanol, and safranin successively to a bacterial smear, allowing for color fixation and differentiation. The Gram staining was further confirmed by KOH assay as follows: fresh bacteria were spread on clear glass slides and were treated with a solution of 3% KOH and mixed properly. The development of a mucoid thread indicated that the bacterium is Gram negative while its absence indicated that the bacterium is Gram positive. The Catalase test was performed by mixing one drop of 3% H_2_O_2_ with freshly grown bacterial cultures on a slide. Gas bubbles development indicated catalyse activity [36]. The carbohydrate fermentation assay was done according to the methodology proposed by Aneja [37], while the hydrogen sulfide (H_2_S) test was carried out as described by Warren et al. [38]. An oxidase test was performed as described by Hayward [39] and the development of dark purple color within a half minute confirmed the positive result. According to Hugh and Leifson [40], an oxidative fermentation test was performed, while NO^3−^ reduction test and gelatin hydrolysis activity were performed by following the methodology used by Thankamani and Dev [41]. Lastly, fluorescence emission was observed using King’s B medium by following the standard protocol of Howell and Stipanovic [42].

### Molecular characterization of bacterial isolates

The genomic DNA of all the bacterial strains was extracted using GeneJet Genomic DNA Isolation Kit (@Thermo Scientific Waltham, USA) according to the mentioned protocol. The 16S rRNA genes of the bacterial strains were amplified using universal primer pair 27F (5´ -AGAGTTTGATC-MTGGCTCAG- 3´) and 1492R (5´ -GGTTACCTTGTTAC-GACTT- 3´), respectively by PCR. The amplified PCR products were visualized under a UV transilluminator and were purified from the bands (approx;1500 bp) using Gel and PCR Clean-Up System (Promega, USA). The bacterial species were determined by 16S rRNA genes sequencing. The obtained forward and reverse sequences were joined together in the DNASTAR program. The final sequences were BLAST to retrieve the identical bacterial sequences. All the sequences were then aligned in CLUSTALW. The phylogenetic tree was made using MEGA X program (version 10.1.7) with 1000 bootstraps. The Neighbor-Joining method was followed to study the evolutionary relationships between our bacterial isolates sequences and all the retrieved sequences [23].

### Pathogenicity and seed germination study of potential rhizobacterial strains

Pathogenicity and effect of rhizobacterial isolates namely, Hyd-01F, Hyd-13Z, Gb-T23, St-149D, and Ft-G43 on seed germination were studied by following the paper towel method of Sudisha et al. [43]. In this assay, five tomato seeds (var: Rio grande) were surface cleaned with 1% NaOCl for 2 min followed by washing 3 times in dH_2_O and dried on blotter paper. Tomato seeds were then treated with 0.1% sterilized CMC as an adhesive material followed by dipping in 30 mL of each bacterial suspension containing 1 × 10^7^ cfu/mL for 2 h. Seeds treated with distilled water only were control treatments for comparison. Bacterized seeds (25 seeds) were then aseptically placed on a moist double-layered paper towel in trays. All the trays were placed at 28 ± 2 °C for 15 days. There were three trays per treatment. After 07 days, seed germination percentage (*S*_*GP*_) was recorded by using the formula:

$$Seed\;Germinaion\;\%=\frac{No.\;of\;germinated\;seeds}{Total\;No.\;of\;seeds}X100$$

Data on plumule and radical length (cm) and disease were recorded 15 days after treatment. The Seedling Vigor index (S_VI_) was calculated by following the formula:

$$\text{SVI}=(\mathrm{mean}\;\mathrm{root}\;\mathrm{length}+\mathrm{mean}\;\mathrm{shoot}\;\mathrm{length})\times\%\;\mathrm g\mathrm e\mathrm r\mathrm m\mathrm i\mathrm n\mathrm a\mathrm t\mathrm i\mathrm o\mathrm n$$

### Effect of rhizobacteria and GPE applications on *EB* disease and plant growth promotion

A repeated pot experiment was set up to study the effect of individual and combined application of bacterial isolates (Hyd-01F, Hyd-13Z, Gb-T23, St-149D, and Ft-G43) and GPE on suppressing *EB* disease incidence and plant growth promotion by following the procedure of Rasool et al. [2]. Tomato seeds (vir; Rio grande) were surface cleaned with 1% NaOCl, washed 3 times in dH_2_O, and blotter dried before bacterization. Seeds were treated with 0.1% CMC as an adhesive material followed by dipping in 30 mL of individual bacterial suspensions for 2 h. Five bacterized seeds were then sown in plastic pots (10 L) containing 8 kg of sterilized potting mixture i.e., sand: clay: farm yard manure at the rate of 1:1:1 [44]. At four weeks-old seedlings, rhizospheric soil was flooded with 15 mL bacterial suspension individually. The conidial suspension was formulated from a week old *A. solani* culture grown on a PDA medium. For this, an actively growing culture of*A. solani* was flooded with distilled water and Shaked well to free the conidia from mycelial mates, filtered through muslin cloth, and conidial concentration was kept 1 × 10^6^ conidia ml^–1^ with the help of a hemocytometer[45]. After 1 day of soil flooding with bacterial isolates, tomato seedlings were treated with *A. solani* conidial suspension until run-off with the help of a hand-held sprayer [13]. Relative humidity (~ 70%) required for *EB* disease development was maintend by spraying the plants with distilled water.

Ginger powder extract (25 g/L) was prepared in distilled water and applied as a foliar application on individual and combined treatments along with bacterial isolates. Plants sprayed with Antracol (70% WP) at 0.2% level served as a positive control, while the plants inoculated with fungal conidial suspension alone were kept as a negative control. Pots were placed under controlled conditions and other agronomic activites were kept the same for all the treatments. The experiment was conducted under CRD design with 4 repeats. The treatments include; T1 = Hyd-01F + *A. solani;* T2 = Hyd-13Z + *A. solani;* T3 = Gb-T23 + *A. solani;* T4 = St-149D + *A. solani;* T5 = Ft-G43 + *A. solani;* T6 = Bacterial consortia + *A. solani;* T7 = GPE + *A. solani;* T8 = Hyd-01F + GPE + *A. solani;* T9 = Hyd-13Z + GPE + *A. solani;* T10 = Gb-T23 + GPE + *A. solani;* T11 = St-149D + GPE + *A. solani;* T12 = Ft-G43 + GPE + *A. solani;* T13 = Bacterial consortia (Detail of consortia) + GPE + *A. solani;* T14 = Antracol (Positive control); T15 = Negative control.

Data on disease control was recorded three weeks after the treatment applications while data on plant growth traits (seed germination, plomule length, radical length, vigor index, shoot length, root length, fresh shoot weight, dry shoot weight, fresh root weight and dry root weight) was recorded 45 days after transplantation. Disease severity was recorded on 0–4 disease rating scale of Li and Dong [46] while the data on the percent disease index (PDI) was calculated by following the formula of McKinney [47] as given below;

$$PDI=\frac{sum\;all\;disease\;rating}{total\;number\;of\;ratings\;\times\;maximum\;rating}\times100$$

### Chlorophyll contents

Briefly, chlorophyll contents and carotenoids were determined by adopting the procedure of Hiscox and Israelstam [48]. In this test, 1 g of fresh tomato leaf samples was finely chopped into small segments, ground using a pestle and mortar with 100 mL of an 80% acetone solution (v/v), and subsequently filtered through Whatman No. 1 filter paper. The resulting solution, comprising 100 mL in 80% aqueous acetone, was prepared. The optical density of the prepared mixture was measured using a spectrophotometer at 649 nm and 665 nm wavelengths. Chlorophyll concentration in leaf samples were calculated using the formulas of Holm-Hansen and Riemann [49] given below;


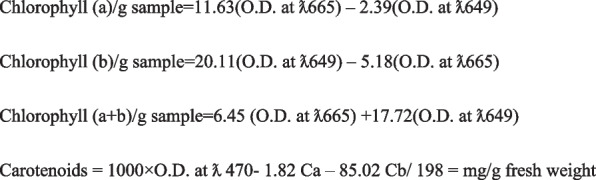


Where;

Ca = Concentration of chlorophyll (a)

Cb = Concentration of chlorophyll (b)

### Defense-related enzymes in tomato plants

Metabolic and biochemical indicators for plant resistance against *A. solani* were determined in leaves samples collected from 60 days old plants.

#### Total phenolics

The Folin-Ciocalteu method was followed to determine the total phenolics as per Rasool et al. [2]. In this test, 1 g fresh tomato leaf samples were mixed in 10 mL ethanol (80%) and agitated at 70 °C for 15 min. After this, 200 μL extract was mixed with Folin Ciocalteau reagent (500 μL) and placed at 25 °C for 3 min. To this solution, 800 μl/0.8 ml of 7.5% saturated Na_2_CO_3_ was added followed by incubation for 30 min at 45 °C. Absorbance was taken using a UV–Visible spectrophotometer at 765 nm against a blank. The total phenolic contents were recorded against the standard curve reference number.

#### Total protein contents

A modified method of Khan et al. [50] was used to determine the total protein contents. In this test, 0.5 g fresh leaf samples were mixed in 10 mL cold Na_3_PO_4_ buffer (100 mM; pH 7.4). The prepared mixture was stirred at 12,000 rpm for 15 min at 4 °C and the final supernatant of crude enzyme extract was obtained. The total protein contents were quantified spectroscopically using bovine serum albumin as a standard.

#### Peroxidase (PO)

Peroxidase activity test was performed by adopting the methodology of Hammerschmidt et al. [51]. In this assay, PO activity was determined by using solution containing enzyme extract (0.5 mL), 1.5 mL of pyrogallol (0.05 M) and 0.5 mL H_2_O_2_ (1%) followed by incubation at room temperature. Change in absorbance was recorded spectrophotometrically at 420 nm wavelength at 30 s intervals for 3 min against a standard. The PO activity was measured as Katal/mg of the total proteins.

#### Polyphenol oxidase (PPO)

The methodology of Hyder et al. [52] was followed to quantify the Polyphenol oxidase activity (PPO) as an indicator of defense induction against *A. solani*. The assay was performed by preparing a mixture of crude enzyme extract (200 mL) and 1.5 mL of 0.1 M Na_3_PO_4_ buffer. In the reaction mixture, 200 mL of 0.01 M catechol was loaded to initiate reaction and analyzed spectroscopically at a wavelength of 495 nm.

#### Phenylalanine ammonia lyase (PAL)

The activity of Phenylalanine ammonia-lyase was determined according to the methodology of Whetten and Sederoff [53]. The reaction mixture was prepared by adding 100 mL of the enzyme, 500 mL of 50 mM Tris HCL, and 600 mL of 1 mM L-phenylalanine and incubation for 1 h. The reaction was stopped by adding 2N HCL to the reaction mixture, followed by adding toluene (1.5 mL) in it, vortex for 30 s, and centrifugation (1000 rpm) for 5 min. Toluene fraction was separated and toluene phase was determined spectrophotometrically at 290 nm against the toluene as blank. Standard curve was constructed using cinnamic acid in toluene. PAL reaction was represented as Katal/mg of total proteins.

#### Catalase (CAT) activity

The catalytic activity was determined spectrophotometrically in two phases by following the methodology of Dhindsa et al. [54]. The reaction mixture contains 400 μL of 5% H_2_SO_4_, enzyme extract (100 μL), and 1 mL of 0.1 M H_2_O_2_. In the reaction mixture-1 (R_m_1), H_2_SO_4_ was added along with the other reagents and the mixture was vortexed at 26 ◦C for 30 s before taking the readings while in the case of mixture-2 (R_m_2), H_2_SO_4_ was added after vertexing reaction mixture at 26 ◦C for 1 min. The absorption was taken @ 270 nm using a spectrophotometer. The difference (R_m_1—R_m_2) represented catalase activity which was shown in Unit; min^−1^ g ^−1^ of protein () (reference number).

### Statistical analysis

Data was analyzed in Statistix 8.1 and MS Excel 365. Experiments were conducted in CRD with three replicates. Mean values were calculated, and means were compared by ANOVA using LSD @ 5% (*P* ≤ 0.05). The correlation analysis was performed in MS Excel 365.

## Results

### Inoculum and morphological characterization of *A. solani*

A total of six fungal isolates (*As-1*, *As-2, As-3, As-4, As-5,* and *As-6*) were recovered from the collected symptomatic tomato plant samples on PDA media containing Petri plates. All the recovered isolates of *A. solani* displaying the highest disease virulence were identified based on peculiar morphological features. Fungal isolates displayed dark brown, irregular colonies with aerial mycelial growth. Morphological features i.e., horizontal septation (6.96 to 7.93 µm), vertical septation (1.50 to 2.22 µm), conidia length (174.2 to 187.6 µm), conidial width (14.09 to 16.52 µm), beak length (93.06 to 102.26 µm), and sporulation is presented in Table 1.

**Table 1:** Morphological featuring of *A. solani* associated with EB disease in tomato

Morphological Features / Fungal Isolates	*As-1*	*As-3*	*As-5*
Colony Margins	Irregular	Irregular	Irregular
Colony color	Dark Brown	Dark Brown	Dark Brown
Mycelial growth	Aerial	Aerial	Aerial
Sporulation	Positive	Positive	Positive
Horizontal Septations	7.93 ± 0.15 µm	8.04 ± 0.14 µm	6.96 ± 0.11 µm
Vertical Septations	1.99 ± 0.08 µm	2.22 ± 0.14 µm	1.50 ± 0.11 µm
Length of Conidia	180.60 ± 1.12 µm	187.60 ± 1.32 µm	174.20 ± 1.70 µm
Width of Conidia	15.12 ± 0.51 µm	16.52 ± 0.27 µm	14.09 ± 0.52 µm
Beak Length	98.37 ± 0.96 µm	102.26 ± 1.08 µm	93.06 ± 0.87 µm

Presented Means values are the average of ten independent readings; ± represents standard error (SE) values

### Virulence confirmation of *A. solani*

**Fig. 1:**
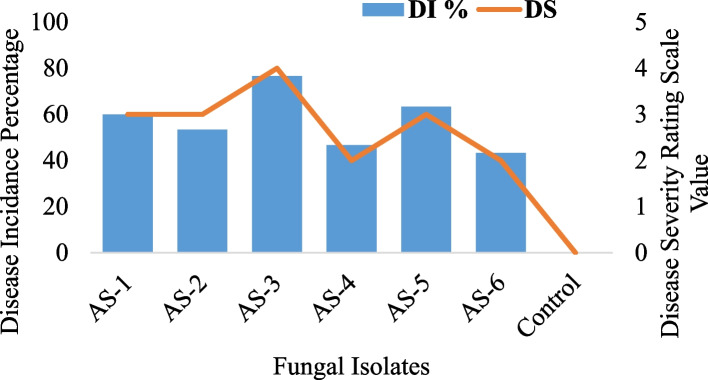
Pathogenicity assay to confirm the virulence of *A. solani* on tomato plants under controlled conditions. DI % = Disease Incidence percentage; DS = Disease Severity on Disease Rating Scale

A total of six *A. solani* isolates were subjected to virulence confirmation on tomato plants (Variety; Rio Grandy) in a detached leaf assay. All the tested isolates showed variability in response to producing characteristic *EB* symptoms and the results are presented in Fig. 1. Among all the fungal isolates, *As-3* produced the highest disease incidence-*DI* of 76.7% followed by *As-5* (63.3%) and *As-1* (60%) while the lowest disease incidence was shown by *As-6* (43.3%). On a disease rating scale, *As-3* showed the highest disease severity-*DS* (4; 51–75% leaf area infected) while *As-1, As-2*, and *As-5* showed *DS* (3; 26–50% leaf area infected).

### Biochemical assay

Bacterial isolates with strong antagonistic ability were subjected to various biochemical tests and the results are presented in Table 2. Four bacterial isolates viz., Hyd-01F, Hyd-13Z, and Gb-T23 showed positive test results for Gram staining while St-149D and Ft-G43 showed a negative reaction. The Gram stain results were confirmed with the KOH test. The result of the Catalase test reveals that all the bacteria were catalase positive. Additionally, the Carbohydrate fermentation test was positive for Hyd-13Z, St-149D, and Ft-G43 while the test was negative for Gb-T23. All the bacterial isolates except Hyd-01F showed positive results for the Hydrogen Sulphide production test whereas, all the bacterial isolates showed a positive response toward the Oxidase test. In addition, Hyd-13Z, Gb-T23, and St-149D were positive for the Oxidative fermentative test whereas the test was not done for Ft-G43. All bacterial strains displayed positive test results for NO^3−^ reduction and Gelatin hydrolysis. Of all the tested bacterial strains, Hyd-01F, Hyd-13Z, and St-149D revealed negative test results for Fluorescence emission-*FLE* while Gb-T23 and Ft-G43 reflected positive test results.

**Table 2:** Biochemical characterization of bacterial isolates recovered from tomato rhizosphere

**Biochemical Assay**	*Hyd-01F*	*Hyd-13Z*	*Gb-T23*	*St-149D*	*Ft-G43*
GS	+	+	+	-	-
KOH	-	-	-	+	+
CAT	+	+	+	+	+
CF	ND	+	-	+	+
HS	-	+	+	+	+
OXD	+	+	+	+	+
OXFT	-	+	+	+	ND
NR	+	+	+	+	+
GH	+	+	+	+	+
FLE	-	-	+	-	+

+ Positive response;—Negative response, *ND* Not done, *GS* Gram Staining, *KOH* Potassium Hydroxide test, *CAT* Catalase, *CF* Carbohydrate Fermentation, *HS* Hydrogen Sulphide, *OXD* Oxidase test, *OXFT* Oxidative Fermentation, *NR* Nitrate Reduction, *GH* Gelatin Hydrolysis, *FLE* Fluorescence Emission

### Isolation and compatibility among the bacterial strains and GPE

Five independent soil samples collected from the tomato rhizosphere were used to recover 23 bacterial isolates displaying variation in colony characters and color. Based on in vitro dual culture assay against the most virulent strain of *A. solani*, 05 potential bacterial isolates viz., Hyd-01F, Hyd-13Z, Gb-T23, St-149D, and Ft-G43 were selected and subjected to compatibility study among the bacterial strains and also with the GPE at 10%, 15%, and 20% concentration levels. The collected results showed that none of the rhizobacterial strains were inhibited by each other, suggesting that all the bacterial strains were compatible if used in consortium form. Compatibility studies of GPE and rhizobacterial antagonists indicated that the GPE at all concentration levels were also compatible with bacterial isolates.

### Effect of individual and combined application of rhizobacteria and GPE on the mycelial growth of *A. solani in-vitro*

A total of 23 bacterial isolates were studied for their antagonistic potential again the highly virulent strain of *A. solani* (*As-3*). Out of all the tested bacteria, 05 (22%) isolates were found highly effective antagonists against *A. solani*. All five bacterial isolates individually and in a combination with GPE showed a varied response in suppressing the mycelial growth of *A. solani*. Among all the bacterial isolates, St-149D showed highest mycelial inhibition (56.3%) of *A. solani* followed by Hyd-13Z (48.3%) and Gb-T23 (42.0%) over the untreated control. However, the combined application of rhizobacterial isolates and GPE resulted in maximum mycelial inhibition of *A. solani*. Among all the combinations, St-149D + GPE resulted in the highest percentage of mycelial growth inhibition (76.9%) followed by Hyd-13Z + GPE (67.6%) while Ft-G43 + GPE showed 46.6% mycelial growth inhibition over untreated control. Individual application of GPE showed 46.6% mycelial growth inhibition of *A. solani* (Table 3).

**Table 3:** Effect of individual and combined applications of rhizobacteria and GPE on the mycelial inhibition of *A. solani*

Treatments	Average	PIOC
Hyd-01F	5.0 ± 0.15^b^	37.4%
Hyd-13Z	4.1 ± 0.26 ^bcd^	48.3%
Gb-T23	4.6 ± 0.32^bc^	42.0%
St-149D	3.5 ± 0.27^de^	56.3%
Ft-G43	4.8 ± 0.34 ^bc^	39.9%
Hyd-01F + GE	4.0 ± 0.55^ cd^	49.2%
Hyd-13Z + GE	2.6 ± 0.38^ef^	67.6%
Gb-T23 + GE	4.1 ± 0.26 ^bcd^	47.9%
St-149D + GE	1.8 ± 0.22^f^	76.9%
Ft-G43 + GE	4.2 ± 0.20 ^bcd^	46.6%
Ginger Extract	4.2 ± 0.26^bcd^	46.6%
Control	7.9 ± 0.32^a^	0.0%
LSD	0.9092	

*PIOC* Percentage Mycelial Growth Inhibition over Untreated Control

### Molecular identification of isolated bacterial strains

**Fig. 2:**
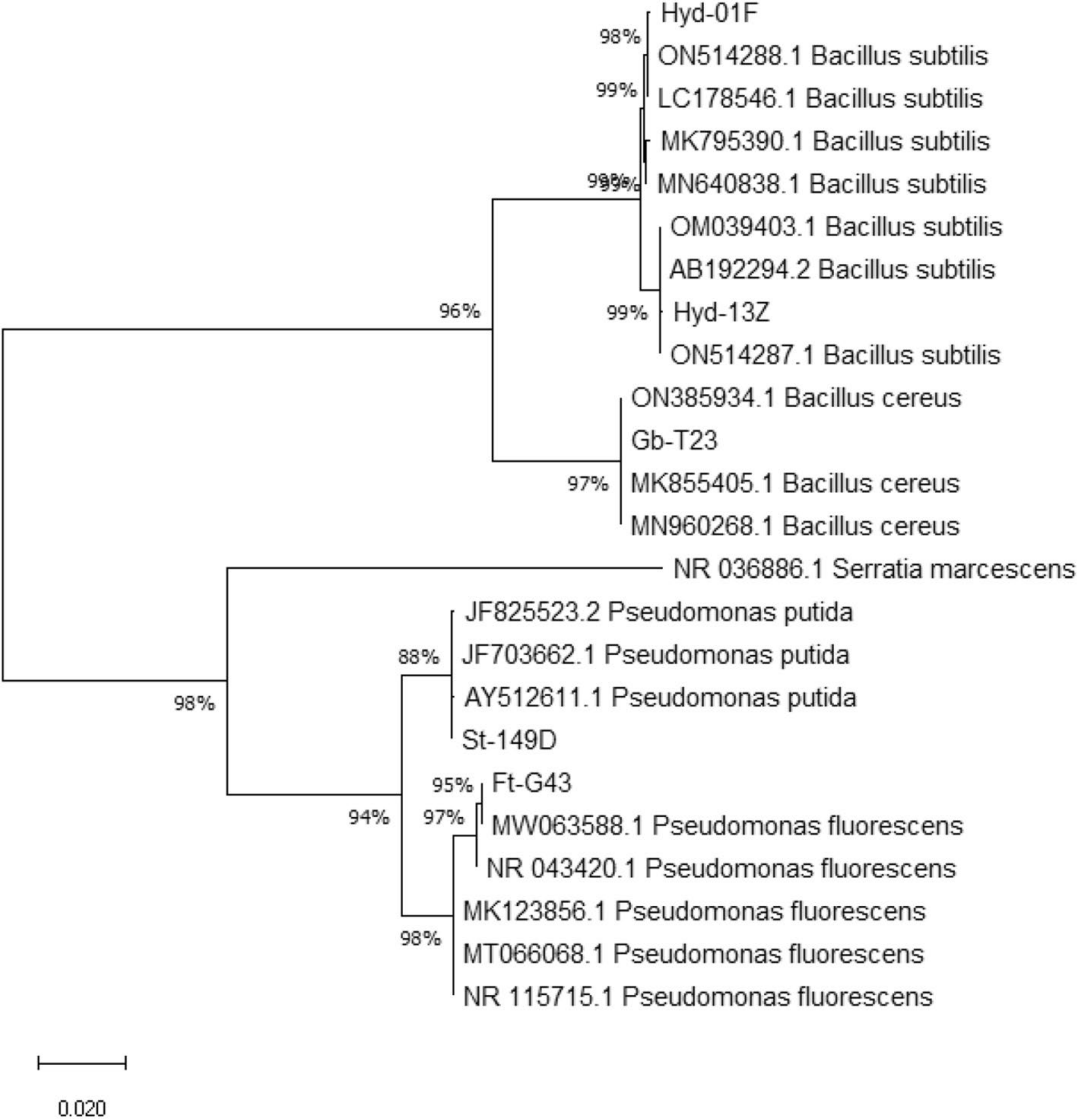
Phylogenetic analysis on the basis of 16S rRNA sequences (approx.1500 bp) displaying relationships between the sequences of representative bacterial isolates and closely related strains. The closely related sequences of bacterial strains were retrieved from NCBI (https://www.ncbi.nlm.nih.gov/)

Rhizobacterial isolates reflecting antagonistic potential were characterized based on 16S ribosomal DNA gene partial sequencing. The bacterial sequences of our isolates (≈ 1500 bp) along with corresponding reference bacterial isolates are displayed in Table 4. It is reflected in the phylogenetic tree that bacterial strains Hyd-01F and Hyd-13Z showed 99.9% identity with *Bacillus subtilis* accessions LC178546 and AB192294 (Fig. 2). Similarly, bacterial strains Gb-T23, St-149D, and Ft-G43 showed maximum sequence similarity with *B. cereus*, *Pseudomonas putida,* and *Pseudomonas fluorescens* respectively.

**Table 4:** Sequence identity of 16S rRNA gene from rhizobacteria and their sequence similarity with the reference bacterial strains

Isolate ID	Sequence (bp)	Accession No	Identified As	Identify with the Accession No	Similarity Index
Hyd-01F	1508	ON891846	*Bacillus subtilis*	LC178546	99.9%
Hyd-13Z	1513	ON878096	*Bacillus subtilis*	AB192294	99.9%
Gb-T23	1498	ON892078	*Bacillus cereus*	ON385934	100%
St-149D	1370	ON892115	*Pseudomonas putida*	JF825523	100%
Ft-G43	1509	ON892085	*Pseudomonas fluorescens*	NR_043420	99.9%

### In vitro assessment of pathogenicity and effect of rhizobacterial strains on tomato seed germination and vigor index

The results of the pathogenicity assay revealed that all the bacterial strains were non-pathogenic and did not produce symptoms in the tomato seedlings. All the bacterial agents improved the tomato seed germination ranging from 85.0 ± 3.54 to 93.2 ± 1.97% over untreated control 76.2 ± 2.39. Our tested bacterial strains significantly improved the plumule (9.60 ± 0.47 to 10.45 ± 0.40%) and radical length (3.25 ± 0.12 to 4.28 ± 0.22%) in tomato seedlings as compared to untreated control, where the plumule and radical length were recorded 7.80 ± 0.39 and 3.40 ± 0.12% respectively. A significant increase in seedling vigor index is also noticed over untreated control as presented in Table 5.

**Table 5:** Pathogenicity conformation of PGPR and their impact on seed quality traits

Bacterial Strains	Germination %	Plumule Length (cm)	Radical Length (cm)	Vigor Index
Hyd-01F	85.0 ± 3.54^b^	10.15 ± 0.98^a^	3.55 ± 0.27^b^	1164.5
Hyd-13Z	88.7 ± 3.15^ab^	10.03 ± 0.54^a^	3.25 ± 0.12^b^	1177.9
Gb-T23	90.0 ± 2.04^ab^	9.80 ± 0.16^a^	3.48 ± 0.29^b^	1195.2
St-149D	93.2 ± 1.97^a^	10.45 ± 0.40^a^	4.28 ± 0.22^a^	1372.8
Ft-G43	90.0 ± 2.04^ab^	9.60 ± 0.47^a^	3.40 ± 0.12^b^	1170.0
UTC	76.2 ± 2.39^c^	7.80 ± 0.39^b^	2.20 ± 0.30^c^	762.0
**LSD**	**7.7054**	**1.6307**	**0.6893**	-

*UTC* Untreated control

### Testing of rhizobacterial strains and plant extract on early blight disease control and plant growth promotion

The individual and co-inoculated effect of rhizobacterial strains and GPE on seed germination, *EB* disease suppression, and growth promotion in tomato plants was examined in the presence of *A. solani* (*AS-3*) in a repeated pot trial. Our results showed that tomato seeds treated with individual rhizobacteria and in combination with the GPE showed varied responses on *EB* disease suppression and seed germination as presented in Table 6. Seeds treated with bacterial consortia + GPE showed the highest disease suppression percentage of 78.1% followed by St-149D + GPE (72.2%) and Hyd-13Z + *GPE* (67.5%) while the response of individual application of rhizobacterial applications was ranged from 30.5 to 66.2%. The individual application of bacterial consortia and GPE showed disease suppression percentages of 57% and 51.1%, respectively, and were less effective than Antracol as standard control (69.6%). In the case of tomato seed germination, all the tested bacterial strains alone, in consortia form, and combination with GPE significantly improved the seed germination. Maximum seed germination percentage was recorded in the bacterial consortia + GPE (95.0 ± 2.04) followed by St-149D + GPE (92.5 ± 1.44) and Hyd-13Z + GPE (90.0 ± 2.04) over the control (73.8 ± 2.39) and chemical control as standard treatment (90.0 ± 2). The individual applications of rhizobacterial strains in the presence of *A. solani* showed seed germination ranging from 79.3 ± 1.49 to 88.8 ± 1.25 percent. The seed treatment with GPE individually displayed 79.5 ± 2.1 percent seed germination while bacterial consortia showed 87.5 ± 3.23 percent tomato seed germination in the presence of *A. solani*. ( disease percent incidence is minimum in Antracol treatment but percent disease control in maximum in the bacterial consortia + GPE).

**Table 6:** Effect of rhizobacteria and GPE applications on *EB* disease suppression and plant growth improvement in tomato

Treatments	GP	DIP	PDC
Hyd-01F + *A. solani*	79.3 ± 1.49 fg	27.5 ± 1.71bcd	53.6
Hyd-13Z + *A. solani*	82.5 ± 2.5ef	22.5 ± 1.32efg	62.0
Gb-T23 + *A. solani*	83.0 ± 3.14def	28.8 ± 1.49bc	30.5
St-149D + *A. solani*	88.8 ± 1.25bcd	20.0 ± 1.29fghi	66.2
Ft-G43 + *A. solani*	80.0 ± 0.82f	30.8 ± 1.25b	48.1
Bacterial consortia + *A. solani*	87.5 ± 3.23bcde	25.5 ± 1.32cde	57.0
Ginger Powder extract (GPE) + *A. solani*	79.5 ± 2.1 fg	29.0 ± 1.58bc	51.1
Hyd-01F + GPE + *A. solani*	80.5 ± 2.1f	23.8 ± 1.84def	59.9
Hyd-13Z + GPE + *A. solani*	90.0 ± 2.04abc	19.3 ± 1.38ghi	67.5
Gb-T23 + GPE + *A. solani*	85.0 ± 2.04cdef	22.0 ± 2.12efgh	62.9
St-149D + GPE + *A. solani*	92.5 ± 1.44ab	16.5 ± 0.65ij	72.2
Ft-G43 + GPE + *A. solani*	81.8 ± 2.36ef	22.5 ± 1.55efg	62.0
Bacterial consortia + GPE + *A. solani*	95.0 ± 2.04a	13.0 ± 1.29j	78.1
Antracol	90.0 ± 2.04abc	18.0 ± 1.47hi	69.6
Control	73.8 ± 2.39 g	59.3 ± 1.49a	0.0
**LSD**	**6.1459**	**4.2288**	

*GP* Germination percentage, *DIP* Disease incidence percentage, *PDC* percentage disease control

All the tested variables alone and in combination with GPE significantly influenced the plant growth parameters in the presence of virulent *A. solani* inoculum (Table 7). Among all the treatments, bacterial consortia + GPE and St-149D + GPE significantly increased the shoot length to 141.3 ± 1.75 and 138.3 ± 0.85 respectively. Tomato shoot length ranged from 109.3 ± 2.66 to 122.8 ± 2.39 in individual treatments of bacterial strains while the shoot length ranged from 123.8 ± 1.8 to 138.3 ± 0.85 in the treatments of bacterial strains in combination with GPE. Of all the treatments, GPE showed the minimum increase in shoot length (113.0 ± 1.68). In the case of root length, bacterial consortia + GPE significantly increased the root length (23.0 ± 1.68) as compared to individual applications of bacterial strains and in combination with GPE where the root length was recorded ranging from 15.8 ± 1.75 to 20.3 ± 1.25 and 16.3 ± 1.11 to 21.5 ± 0.65 respectively. GPE alone was observed as least effective in increasing the root length (15.3 ± 2.39).

**Table 7:** Effect of individual bacterial inoculants and in combination with GPE on plant growth promotion

Treatments	SL (cm)	RL (cm)	FSW (g)	DSW (g)	FRW (g)	DRW (g)
Hyd-01F + *A. solani*	118.8 ± 2.02def	15.8 ± 1.75de	184.8 ± 3.01 g	21.8 ± 1.65jk	2.4 ± 0.09efg	0.93 ± 0.11 cd
Hyd-13Z + *A. solani*	109.3 ± 2.66 g	16.0 ± 1.08cde	188.8 ± 2.14 fg	22.5 ± 1.94ijk	2.3 ± 0.13gh	0.93 ± 0.09 cd
Gb-T23 + *A. solani*	115.5 ± 2.75 fg	15.8 ± 2.14de	181.5 ± 3.88 g	21.0 ± 1.96kl	2.5 ± 0.21efg	0.98 ± 0.06 cd
St-149D + *A. solani*	122.8 ± 2.39cde	20.3 ± 1.25abcd	205.3 ± 1.93e	27.0 ± 1.47fgh	2.9 ± 0.11 cd	1.15 ± 0.06bcd
Ft-G43 + *A. solani*	116.5 ± 3.4ef	17.3 ± 1.55bcde	193.5 ± 4.29f	24.8 ± 1.44hijk	2.3 ± 0.14 fg	1.03 ± 0.13 cd
Bacterial consortia + *A. solani*	127.0 ± 1.47bc	19.5 ± 1.85abcde	211.8 ± 2.06bcde	29.5 ± 0.65efg	2.8 ± 0.13de	1.23 ± 0.05bc
Ginger Powder extract (GPE) + *A. solani*	113.0 ± 1.68 fg	15.3 ± 2.39ef	207.3 ± 3.33de	26.3 ± 1.65ghi	2.5 ± 0.15efg	1.10 ± 0.09bcd
Hyd-01F + GPE + *A. solani*	123.8 ± 1.8bcd	17.5 ± 1.19bcde	208.8 ± 2.39cde	25.8 ± 1.11ghij	2.7 ± 0.14def	1.15 ± 0.19bcd
Hyd-13Z + GPE + *A. solani*	128.0 ± 1.78bc	16.5 ± 1.71cde	215.3 ± 1.03bc	32.3 ± 1.31cde	3.0 ± 0.06 cd	1.13 ± 0.13bcd
Gb-T23 + GPE + *A. solani*	129.0 ± 2.08bc	16.3 ± 1.11cde	212.0 ± 2.68bcde	30.5 ± 1.44def	2.9 ± 0.11 cd	1.03 ± 0.05 cd
St-149D + GPE + *A. solani*	138.3 ± 0.85a	21.5 ± 0.65ab	227.5 ± 1.32a	39.8 ± 1.11a	3.4 ± 0.08ab	1.38 ± 0.06ab
Ft-G43 + GPE + *A. solani*	125.0 ± 2.38bcd	17.8 ± 2.06bcde	214.5 ± 2.99bcd	34.0 ± 1.08bcd	3.2 ± 0.11abc	1.20 ± 0.11bcd
Bacterial consortia + GPE + *A. solani*	141.3 ± 1.75a	23.0 ± 1.68a	228.0 ± 1.68a	38.0 ± 1.47ab	3.6 ± 0.13a	1.55 ± 0.13a
Antracol	130.0 ± 2.86b	20.5 ± 1.94abc	216.8 ± 1.65b	36.0 ± 2.08abc	3.2 ± 0.13bc	1.40 ± 0.20ab
Control	101.8 ± 2.87 h	10.8 ± 0.85f	139.3 ± 2.69 h	17.3 ± 1.31 l	1.9 ± 0.14 h	0.90 ± 0.07d
LSD	6.4788	4.6173	7.4843	4.2474	0.3681	0.3170

*SL* Shoot length, *RL* Root length, *FSW* Fresh shoot weight, *DSW* Dry shoot weight, *FRW* Fresh root weight, *DRW* Dry root weight

Tomato plants treated with bacterial isolates alone and in combination with GPE sustained *A. solani* induced decrease in root and shoot fresh weight and dry weight (Table 2). The maximum fresh and dry shoot weights (228.0 and 38.0 g respectively) were recorded for the plants treated with a combined application of bacterial consortia and GPE in the presence of *A. solani*. Among the individual bacterial treatments in the presence of *A. solani*, fresh and dry shoot weight ranged from 181.5 to 205.3 g and 21.0 to 27.0 g respectively. While fresh and dry shoot weight of plants treated with the combined applications of individual bacterial strains and GPE was recorded at 208.8 to 227.5 g and 25.8 to 39.8 g respectively. In the case of fresh and dry root weight, all the treatments improved the FRW and DRW over the control and standard control as presented in Table 7. Amon all the treatments, *A. solani* inoculated plants treated with the bacterial consortia and GPE significantly increased the FRW (3.6 g) and DRW (1.55 g) as compared to the plants challenged with *A. solani* (control). Plants treated with the bacterial strains + GPE significantly increased the FRW (2.7 to 3.4 g) and DRW (1.03 to 1.38 g) as compared to the individual treatments of bacteria strains (2.3 to 2.9 g) and (0.93 to 1.15 g) respectively.

### Chlorophyll contents

**Fig. 3:**
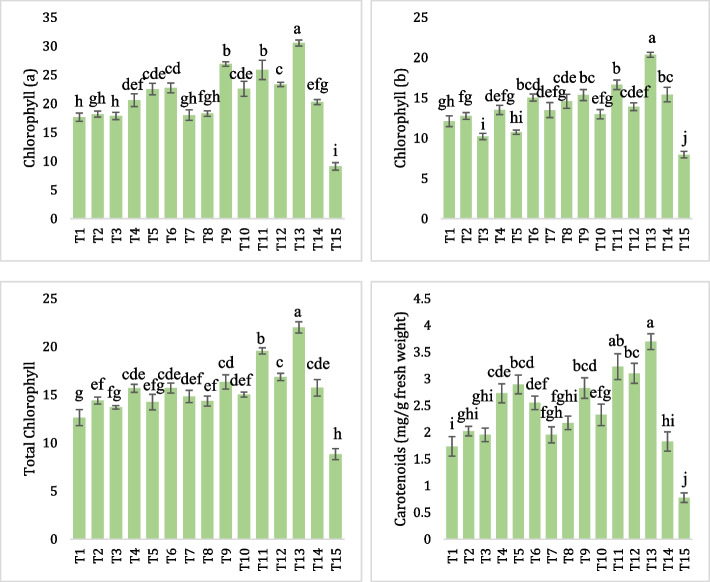
Estimation of Photosynthetic pigments in tomato plants treated with individual bacterial inoculants and in combination with GPE in the presence of pathogenic *A. solani*

The photosynthetic pigments were significantly reduced in the plants challenged with *A. solani* (Fig. 3). All the bacterial treatments individually and in combination with GPE significantly improved the Chlorophyll contents and carotenoids. In the case of Chlorophyll a content, *A. solani* infected plants challenged with bacterial consortia + GPE significantly increased Chlorophyll a (30.525 mg/g) followed by Hyd-13Z + GPE (26.875 mg/g) and St-149D + GPE (25.85 mg/g) while all other treatments including individual applications of PGPR and GPE showed less effectiveness in enhancing the Chlorophyll a content. Similarly, in the case of Chlorophyll b, total Chlorophyll and Carotenoids, *A. solani* infected plants inoculated with a combined application of bacterial consortia and GPE significantly improved the photosynthetic pigments as compared to all other treatments (individual bacterial applications and GPE) in the presence of *A. solani* inocula. On the other side, photosynthetic pigments were observed significantly lower in the plants challenged with *A. solani* (control).

### Defense related enzymes induction in tomato plants

#### Total phenolic contents-*TPC*

**Fig. 4:**
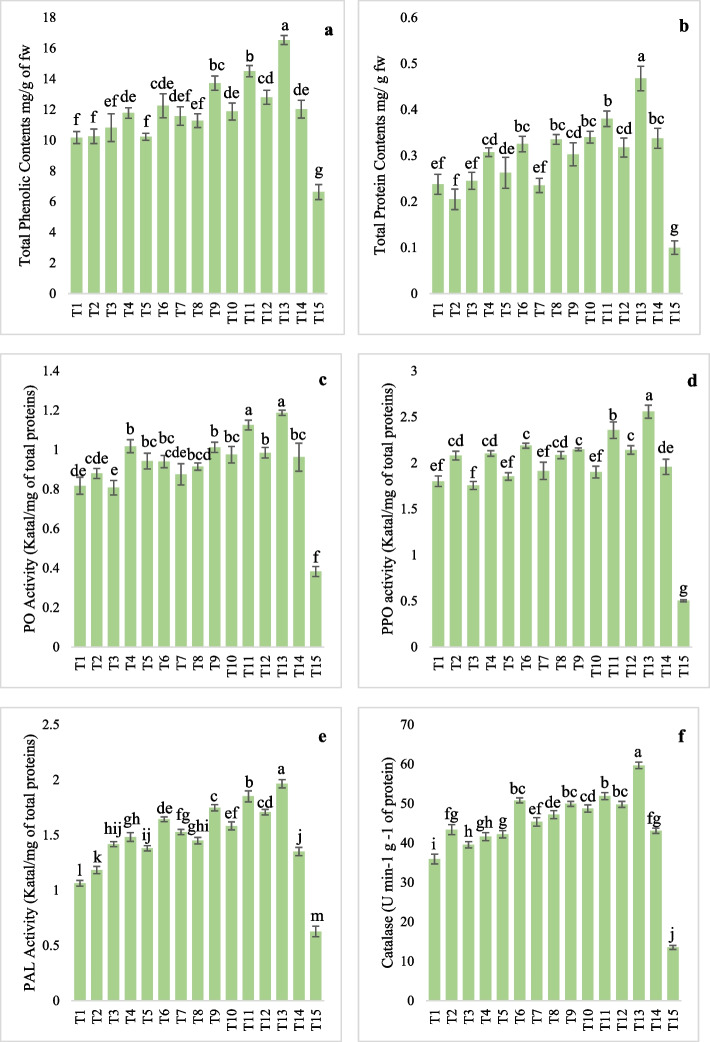
Detection and quantification of defense-related enzyme induction in plants challenged with *A. solani* under influence of individual bacterial inoculants and combined application with GPE. **a** Represent total phenolic contents; (**b**) = Total protein contents; (**c**) = PO; (**d**) = PPO; **e** = PAL and (**f**) = Catalase activity

Tomato plants inoculated with *A. solani* grown under individual and combined applications of bacterial strains and GPE showed a significant increase in TPC over the plants challenged with *A. solani* as a control treatment as presented in Fig. 4a. The phenolic contents were observed highest in the tomato plants' co-inoculation of bacterial consortia + GPE (16.54 mg/g of fresh weight) followed by St-149D + GPE (14.5 mg/g) and Hyd-13Z + GPE (13.73 mg/g) over control treatment (6.63 mg/g fresh weight). The phenolic contents were recorded ranging from 10.18 to 11.78 mg/g fresh weight in plants treated with individual bacterial agentsin the presence of *A. solani*. On the other side, tomato plants individually treated with bacterial consortia (12.25 mg/g of fresh weight) and GPE (11.58 mg/g of fresh weight) produced low TPC as compared to the combined application of bacterial consortia and GPE.

#### Total protein contents

The analyzed data displayed a strong interaction of *A. solani* inoculated plants treated with the individual bacteria strains and in combination with GPE in producing the total protein contents (Fig. 4b). In the case of tomato plants treated with individual bacterial isolates in the presence of *A. solani* showed total protein contents ranged from 0.21 to 0.31 mg/g fresh weight, while the total protein contents were recorded ranging between 0.30 to 0.38 mg/g fresh weight of the plants treated with individual bacterial isolates in combination with GPE. Among all the treatments, bacterial consortia + GPE produced significantly high total protein contents (0.47 mg/g fresh weight) as compared to bacteria consortia (0.33 mg/g) and GPE (0.24 mg/g).

#### Peroxidase activity-PO

All the treatments induced the defense enzymes in the tomato plants over control where only *A. solani* was applied as shown in Fig. 4c. In tomato plants, treated with consortia + GPE, a notable increase in PO activity of 1.19 Katal/mg total proteins was observed followed by St-149D + *GPE* (1.13 Katal/mg of total proteins) and Hyd-13Z + GPE (1.01 Katal/mg total proteins) and individual application of St-149D (1.02 Katal/mg total proteins). PO activity ranged from 0.81 to 1.02 Katal/mg total proteins. Among all the treatments, minimum PO activity (0.81 Katal/mg total proteins) was recorded in the plants inoculated with Gb-T23 bacterial isolates in the presence of *A. solani*.

#### Polyphenol oxidase (PPO)

An increase in PPO activity in tomato leave samples was recorded in all the treatments over the control. The collected data showed that bacterial consortia + GPE application in the presence of *A. solani* showed maximum PPO activity (2.56 Katal/mg total proteins) followed by St-149D + GPE (2.36 Katal/mg total proteins) and Hyd-13Z + GPE (2.15 Katal/mg total proteins) whereas, the minimum PPO induction was recorded in Gb-T23 + GPE (1.90 Katal/mg total proteins). In the case of individual applications of bacterial strains, and GPE, PPO activity ranged from 1.76 to 2.10 Katal/mg total proteins as presented in Fig. 4d.

#### Phenylalanine ammonia-lyase (PAL)

PAL activity was recorded as significantly higher in all the plants inoculated with individual bacterial isolates, consortia, and in combination with GPE in the presence of *A. solani*. PAL activity was high in the bacterial consortia + GPE group (1.97 Katal/mg total proteins followed by St-149D + GPE (1.85 Katal/mg total proteins) and Hyd-13Z + GPE (1.75 Katal/mg total proteins) when compared to positive control treatment (0.63 Katal/mg total proteins). In the case of inoculation with individual bacterial stains, PAL activity ranged from 1.06 to 1.48 Katal/mg of total proteins while individual applications of bacterial consortia and GPE showed PAL production of 1.64 and 1.53 Katal/mg of total proteins respectively (Fig. 4e).

#### Catalase (CAT) activity

Catalase production was recorded as significantly high in all the treatment groups over the control as presented in Fig. 4f. Among all the treatments, bacterial consortia + GPE in the presence of *A. solani* showed the highest CAT production (59.68 U min^−1^ g^−1^ of protein) whereas, in all other bacterial combinations with GPE, CAT production ranged from 47.18 to 51.88 U min^−1^ g^−1^ of protein over the control treatment where CAT production was recorded 13.53 U min^−1^ g^−1^ of protein. In case of individual bacterial treatments in the presence of *A. solani*, CAT activity was observed ranging from 35.95 to 43.38 U min^−1^ g^−1^ of protein. In the case of bacterial consortia in the presence of *A. solani*, CAT activity was recorded at 50.78 U min^−1^ g^−1^ of protein and 45.35 U min^−1^ g^−1^ of protein in the individual GPE group.

## Discussion

The present study investigated the potential of synergistic interactions between rhizobacterial isolates and ginger powder extract (GPE) against Early Blight (*EB*) disease caused by *Alternaria solani* in tomato plants. The individual and combined effects of the discovered bacterial isolates and GPE on suppressing *A. solani* were both studied. The findings of this study provide useful insights into disease control techniques that are both sustainable and environmentally friendly.

A total of twenty-three strains of soil rhizobacteria were tested against *A. solani* to identify and characterize promising biological control isolates. Five strains, identified as *Pseudomonas putida* St-149D, *Pseudomonas fluorescens* Ft-G43, *Bacillus subtilis* Hyd-13Z, *Bacillus subtilis* Hyd-01F, and *Bacillus cereus* Gb-T23 with potential plant growth promoting activities, were found to show enhanced antagonistic activity against *A. solani* infection. The inhibition of mycelial growth in vitro was between 37–57% as compared to control. The inhibitory efficacy of *Pseudomonas* and *Bacillus* against *A. solani* may be attributed to the existence of antibiotic substances such as Surfactin, bacillomycin, zwittermycin A, pyoluteorin, pyrrolnitrin, pyocyanin, iturin, and 2,4-diacetylphloroglucinol [14, 55]. These antibiotic substances can inhibit fungal growth by targeting their cellular processes [56, 57]. Antibiotic substances derived from PGPRs provide a sustainable and ecologically sound substitute for synthetic fungicides. These compounds have the ability to be generated through bacterial proliferation in the rhizosphere and exert a biostimulatory influence on plant development and offer defense against a range of pathogens.

Treatment with GPE achieved mycelial inhibition of 46.6% suggesting it be an effective compound for the control of *A. solani*. Other plant extracts such as *A. indica*, *A. sativum*, *P. lysterophorus*, and *D. stramonium* have been also reported as potential inhibitors of *A. solani* [58]. Besides, several researches have found that GPE possesses a broad spectrum antimicrobial properties and acts as fungicide by preventing spore formation and germination and the distortion of the hyphae of different phytopathogens [59], such as *Fusarium oxysporum*, *Botritys cinerea* [60], *Alternaria alternate* [61], *Fusarium solani* [62], among others. Also, the combination of GPE with the bacteria *Bacillus cereus* Gb-T23 and *Pseudomonas fluorescens* Ft-G43 maintained fungal inhibition compared to treatment with GPE alone, while the combination of this compound with *Bacillus subtilis* Hyd-01F, Hyd-13Z, and *Pseudomonas putida* St-149D has demonstrated an increased antifungal potential. The antimicrobial and inhibitory properties of extracts derived from plants, such as ginger powder extract, have been the subject of numerous studies demonstrating their potential for managing pests and pathogens. It has been demonstrated that the concurrent implementation of these two agents has the potential to exploit their synergistic or complementary interactions in order to efficiently manage fungal diseases of crop plants [63, 64].

Under in vitro conditions, it was found that seed bacterized with the PGPR (St-149D, Ft-G43, Hyd-13Z, Hyd-01F, and Gb-T23) enhanced seed germination and other growth indicators. Similar results were reported under in vitro and controlled conditions with strains of this bacteria genera in tomato plants [65–67] and other vegetable plants [68, 69]. Seed germination depends on environmental conditions and the balance of abscisic acid/gibberellins ratio to promote dormancy-breaking. According to numerous studies, the use of PGPR facilitate seed germination and seedling growth, possibly due to the synthesis of gibberellic acid [69]. However, germinating seeds are vulnerable to living and nonliving factors including pathogen infection[70].

The current study suggests that the application of PGPR in combination with GPE increases tomato seed germination in presence of the phytopathogen and effectively manages the EB disease of tomatoes in a pot trial. However, the germination percentage remained unchanged at 90% when *Bacillus subtilis* Hyd-13Z and GPE were applied in combination. Furthermore, a significant increase was noted when *Pseudomonas putida* St-149D bacteria were combined with GPE (92%).

In addition, a reduction in the percentage of disease incidence was noticed with the later treatment compared to the chemical treatment, achieving a disease suppression of 78.1% suggesting a synergistic action of biocontrol agents and plant products. These results are similar to those of Latha et al. [14] who showed that a talc based mixture of Zimmu leaf extract and PGPR reduced the percent disease severity by 18% over the controls, and also improved seed germination and fruit yield. In this regard, it has been reported that the use of microbial consortia, composed of compatible PGPR, could lead to reduced disease incidence through synergistic action[71], besides, this could be advantageous when dealing with many plant diseases [14].

In addition to eliciting defense against EB disease, all treatments also promoted plant growth, where the application of the bacterial consortium with the GPE obtained the best results in shoot and root length, and their dry and fresh weights. Even though the fungicide application at its recommended dose provided effective disease control however, it poses risk to human health, pesticide resistance, and environmental impact would be potential issues. The combination of biological control agents with synthetic pesticides could both reduce the risk of the emergence of pesticide resistance and improve disease control compared to that obtained applying the biological control agent individually [72]. Various studies have shown that combinations of biological control agents with fungicides are more effective than single treatments. For example, [73] reported that the co-application of difenoconazole with *B. amyloliquefaciens* synergistically increased the efficacy of the fungicide against *Fusarium* wilt. Therefore, the development of biocontrol strategies using PGPR should have both biocontrol and growth-promoting potential for the sustainability of crops, such as tomatoes.

All above ground parts of the tomato plant might be harmed by *EB*. Due to insufficient leaf area, oxidative explosion, and increased activity of chlorophyll-degrading enzymes and chlorophyllase under disease conditions, the plant is unable to absorb light, which slows down the rate of photosynthesis [74]. This was observed in Fig. 3, as a sharp decrease of chlorophyll (a, b) and carotenoids. However, *Alternaria*-infected plants treated with PGPRs alone and in combination with GPE showed an increase in the chlorophyll a, b, total chlorophyll, and carotenoids compared with the Antracol treatment. Likewise, Awan et al. [75] reported the interactive effect of the *B. subtilis* with plant nutrients conferred resistance in the infected tomato plants against EB by altering the chlorophyll contents, carotenoids, and phenolics. This can be attributed to the role of PGPR in N_2_ fixation along with IAA production and phosphate metabolizing tendencies [76].

Stimulation of plant growth by rhizobia could also be due to the suppression of plant diseases. This suppression induced by PGPR may be direct, through inhibition of pathogen growth, or indirect, through the activation of plant defense mechanisms, through the production of several compounds including phenolic [77]. Phenolic compound functions are diverse in plants, ranging from involvement in growth improvement, reproduction, and defense against stressors [78]. There are reports of increased phenolic compounds in the soil in response to the plant defense participating in the *ISR*, repelling pathogens due to their inhibitory actions. Our results revealed that all the treatments showed different responses in total phenol content, but the increase of total phenolic could be explained as a defense mechanism, which may induce resistance, through lignin synthesis to strengthen the plant cell wall and induction of barrier against *A. solani* [68].

Indirectly, PGPR control plant pathogens by activiating plant defense mechanisms through proteins production [5]. In this study, protein contents were also increased in all the treatments. Our findings are aligned to the study of Awan et al. [75]. Furthermore, antioxidant enzyme contribute significantly in plant resistance induction against abiotic and biotic stresses, particularly phenylalanine ammonia-lyase (PAL), peroxidase (PO), polyphenol oxidase (PPO), catalase (CAT), among other enzymes. Our study suggests that the utilization of bacterial isolates with plant extracts may aid in overcoming the infection by increasing the production of defense-related enzymes. Tomato plants treated with the bacterial consortium and GPE presented higher values of PO, PPO, PAL, and CAT in comparison with the positive and negative control [79, 80]

## Conclusion

The present study provide evidence that *P. putida* St-149D, *P. fluorescens* Ft-G43, *B. subtilis* Hyd-13Z, *B. subtilis* Hyd-01F and *B. cereus* Gb-T23 were compatible and effectively inhibited the growth of *A. solani*. The combination of PGPR strains with GPE suppress *EB* disease and improve tomato plant growth. Besides, higher levels of defensive enzymes, and phenolic compounds may have contributed to suppress *EB* infection. Therefore, based on in vitro and pot assays, the combination of the bacterial consortium (Ft-G43, Hyd-13Z, Hyd-01F, Gb-T23 and St-149 D) with GPE is a promising alternative to control *EB* disease in tomatoes.

## Data Availability

The datasets containing bacterial DNA sequances used in the current study deposited and are available in the National Center for Biotechnology Information repository under the link https://www.ncbi.nlm.nih.gov/nucleotide/. The submitted accessions No. are ON891846, ON878096, ON892078, ON892115 and ON892085.
